# De-escalation of empiric broad spectrum antibiotics in hematopoietic stem cell transplant recipients with febrile neutropenia

**DOI:** 10.1007/s00277-020-04132-0

**Published:** 2020-06-17

**Authors:** Lindsey Rearigh, Erica Stohs, Alison Freifeld, Andrea Zimmer

**Affiliations:** Division of Infectious Diseases, Department of Internal Medicine, University of Nebraska Medical Center, 985400 Nebraska Medical Center, Omaha, NE 68198-5400 USA

**Keywords:** Febrile neutropenia, De-escalation, Broad spectrum antibiotic

## Abstract

Febrile neutropenia (FN) is a common serious complication in patients undergoing hematopoietic stem cell transplantation (HSCT) requiring urgent evaluation and initiation of empiric broad spectrum antibiotics (BSA). The appropriate duration of BSA for FN in patients with negative cultures and no identifiable infection remains undefined. We retrospectively analyzed allogenic and autologous HSCT patients with FN and negative infectious work-up at our facility from 2012 to 2018. The early de-escalation group (EDG) included those who had BSA de-escalation to fluoroquinolone prophylaxis at least 24 h prior to absolute neutrophil count (ANC) recovery after the patient was fever-free for at least 48 h. Among 297 patients undergoing their first HSCT who experienced FN with negative infectious work-up, 83 patients were de-escalated early with the remaining 214 in the standard of care group (SCG) whose BSA were continued until ANC was > 500. Duration of broad-spectrum antibiotics was shorter in EDG compared to SCG (3.86 days vs. 4.62 days, *p* = 0.03). Rates of mortality, new infections, and clinical decompensation requiring intensive care unit transfer and/or pressor use within 30 days were all similar between the two groups (0% vs. 0.4% *p* = 1.00, 0% vs. 1.4% *p* = 0.56, 13.2% vs. 8.4% *p* = 0.27). This indicates that it is safe to de-escalate antibiotics prior to ANC recovery, leading to less BSA exposure.

## Introduction

Febrile neutropenia (FN) complicates the course of approximately 60–90% of autologous and allogeneic hematopoietic stem cell transplantations (HSCT) [1–3]. Fever may be the only presenting sign of infection in this patient population as they are unable to mount an appropriate immune response. FN requires urgent evaluation and prompt initiation of empiric broad spectrum antibiotics (BSA) including an anti-pseudomonal beta-lactam [4]. A clinical or microbiological infection is diagnosed in approximately 40–50% of FN episodes with 10–30% consisting of bacteremias [5–7]. Antibiotic management for persistently neutropenic patients who defervesce on BSA without a diagnosis of infection and are otherwise stable continues to represent a clinical dilemma. While BSA are initiated at the time of FN, there is no consensus on when to de-escalate back to standard prophylaxis prior to neutrophil recovery. The 2010 Infectious Diseases Society of America (IDSA) guidelines recommend continuing BSA in patients with FN who defervesce and have no documented infection until absolute neutrophil count (ANC) recovery as a grade B recommendation, while cessation of BSA with resumption of oral fluoroquinolone prophylaxis with defervesce and continued neutropenia was given a grade C recommendation [8]. However, the European Conference on Infections in Leukemia (ECIL) guidelines published in 2013 recommend stopping empiric antibiotics after 72 h in patients who have been hemodynamically stable and afebrile for at least 48 h irrespective of their ANC [9]. ECIL based this recommendation on a number of prior studies in adults and children demonstrating BSA de-escalation while remaining neutropenic was safe [10–14]. This included double-blind, placebo-controlled [11], retrospective and prospective observational studies [10, 13, 14]. Patients included in these studies were mainly experiencing prolonged neutropenia from cytotoxic chemotherapy regimens, but all de-escalated BSA while neutropenic. In general, recurrence of fever was similar in multiple studies [11, 12] with no difference in mortality between empirical treatment of FN and cessation of antibiotics 48 h after defervescence [10, 11, 13, 14]. Most recently, The National Comprehensive Cancer Network (NCCN) updated 2019 recommendations discuss both options of BSA duration, suggesting that it may be appropriate to de-escalate to fluoroquinolone prophylaxis in patients who defervesce, but with no specific guidance on criteria or timing of de-escalation [15].

Following the release of the ECIL guidelines, our center shifted to a strategy of early de-escalation of BSA to fluoroquinolone prophylaxis (levofloxacin 500 mg orally, once daily). Through this retrospective review, we sought to compare the outcomes of early BSA de-escalation to fluoroquinolone prophylaxis versus continuing BSA until ANC recovery in HSCT recipients both before and after this change was instituted at the University of Nebraska Medical Center (UNMC). We hypothesized that early de-escalation would decrease the total duration of BSA use within 30 days of FN, even accounting for reinstating BSA with recurrent fever episodes or new infections, with the aim to demonstrate early de-escalation in the HSCT population is safe with no increase in mortality, critical care transfers, or new infections within 30 days.

## Methods

This study retrospectively reviewed patients ≥ 19 years old who had received an autologous or allogeneic HSCT at UNMC from 2012 through 2018. Included patients underwent their first HSCT during this time period and received BSA for their first febrile neutropenia episode (ANC < 500 cells/mm^3^ with either a single fever > 38.3 °C or 38 °C sustained over 1 h) without identifiable infectious cause. Patients were excluded if they had undergone multiple HSCTs, remained afebrile throughout their neutropenic period, developed FN within 24 h of ANC recovery, or had a microbiologic or clinically documented source of infection (even if the organism was viral or fungal).

Starting in 2014, our practice changed to allow patients with FN to discontinue BSA and initiate levofloxacin prophylaxis with continued neutropenia (ANC < 500 cells/mm^3^), if they maintained hemodynamic stability and became afebrile (temperature was less than or equal to 38 °C) for 48 h or more while on BSA without a documented infection. BSA were defined as empiric therapy for FN, which primarily consisted of monotherapy with an antipseudomonal beta-lactam (most commonly cefepime or piperacillin-tazobactam) with occasional addition of other agents for empirical coverage of resistant organisms. BSA were started at the onset of FN; fluoroquinolones or alternative prophylactic agents for neutropenia were discontinued. Fluoroquinolone prophylaxis was standard of care for allogeneic HSCT during the entire study period and was incorporated for autologous HSCT starting in 2015.

Patients were categorized into early de-escalation (EDG) or standard of care (SCG) groups based on the timing at which BSA were discontinued. EDG represented the cohort of patients who had BSA de-escalated to fluoroquinolone prophylaxis at least one hospital day prior to ANC recovery and after being afebrile for at least 48 h and clinically stable. SCG represented the cohort of patients who had BSA continued until neutrophil engraftment (ANC > 500 cells/mm) despite abatement of fevers, including 13 patients who experienced FN within 48 h of ANC recovery.

Recurrent febrile neutropenia was defined as relapse of fever in patients who had been afebrile for 48 h within 30 days of initial FN episode. Infectious work-up for the initial and recurrent FN included blood cultures and other symptom directed imaging and testing at the discretion of the primary team. BSA duration was defined as the total number of days the patient received BSA within 30 days of initial FN, including re-escalation due to recurrent fever, new infection, or clinical decompensation. Infections identified within 30 days of initial FN were recorded. Infections were identified by a combination of clinical symptoms, physical exam findings, imaging, and/or positive cultures collected either at the time of re-fevering or any time the primary team deemed necessary.

The primary end-end point was number of days on BSA within 30 days of initial FN. Secondary endpoints by day 30 included mortality, re-hospitalization, clinical de-compensation requiring intensive care unit (ICU) transfer and/or pressor support, new infections, and length of stay from FN (LOS). Patient demographics, transplant characteristics, and clinical data were extracted from the electronic heath records.

Descriptive statistics including the Mann-Whitney test were utilized to compare the primary and secondary endpoints between EDG and SCG. Fisher’s exact test was used to compare categorical data within EDG. A *p* value < 0.05 was considered statistically significant. All analyses were done in SAS 9.4. This study was approved by the Institutional Review Board of the University of Nebraska Medical Center.

## Results

**Fig. 1:**
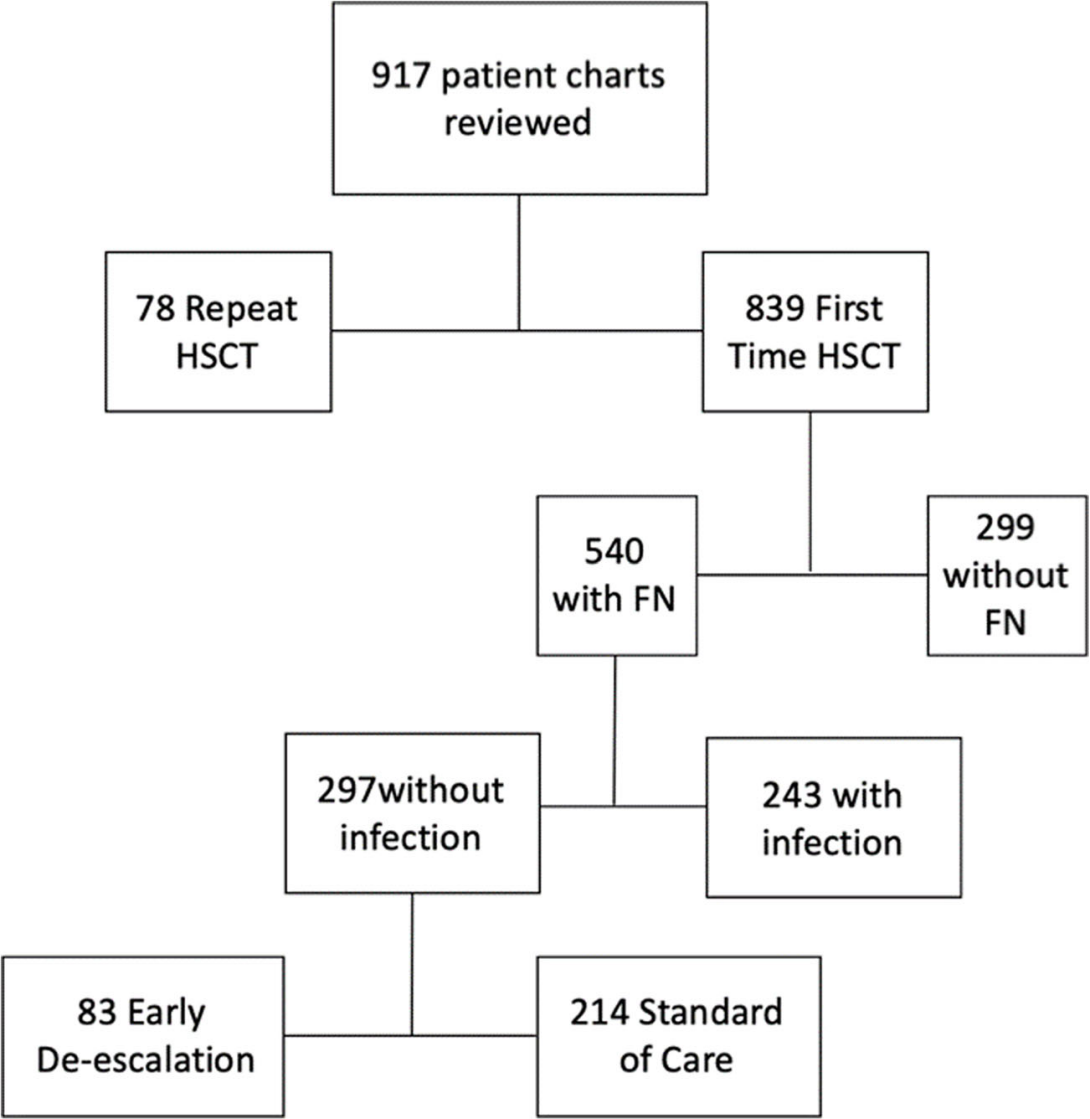
Flow diagram. HSCT: hematopoietic stem cell transplantation; FN: febrile neutropenia

Of 917 HSCT recipients, 297 met inclusion criteria. Treatment was categorized as EDG in 83 patients and as SCG in the other 214 (Fig. 1). Baseline patient characteristics (Table 1) demonstrated EDG contained patients with a higher proportion of allogeneic HSCT, more unmatched transplants, and a higher proportion of acute leukemia or myelodysplastic syndromes as their underlying malignancy. EDG patients were slightly younger and had significantly longer durations of neutropenia at 9.1 days compared to 8 days in SCG (*p* < 0.001). Duration of initial fever was longer in SCG at 3.5 days vs. 2.7 days in EDG (*p* < 0.001).

**Table 1:** Baseline characteristics

	Early de-escalation group	Standard of care group	*P* value
Age (years)	53.7	56.8	0.01
Sex			
Male	59/83 (71%)	130/214 (61%)	0.11
Type of HSCT			
Autologous	47/83 (57%)	183/214 (86%)	
Allogeneic	36/83 (43%)	31/214 (14%)	< 0.001
Type of allogeneic			
Matched	31/36(86%)	31/31 (100%)	
Unmatched	5/36 (14%)	0/31 (0%)	0.06
Underlying malignancy			
AML	13/83 (16%)	11/214 (5%)	0.01
ALL	9/83 (11%)	3/214 (1%)	< 0.001
MDS	7/83 (8%)	8/214 (4%)	0.14
HL	13/83 (16%)	16/214 (7%)	0.05
NHL	27/83 (32%)	86/214 (40%)	0.23
MM	8/83 (10%)	80/214 (38%)	< 0.001
CML	1/83 (1%)	3/214 (1%)	1.00
CLL	2/83 (2%)	0/214 (0%)	0.08
Other	3/83 (4%)	7/214 (3%)	1.00
Conditioning regimen			
Myeloablative	72/83 (87%)	200/214 (93%)	
Reduced intensity	11/83 (13%)	14/200 (7%)	0.1
Duration of neutropenia (days)	9.1	8	< 0.001
Duration of initial fever (days)	2.7	3.5	< 0.001

*HSCT* hematopoietic stem cell transplantation, *AML* acute myeloid leukemia, *ALL* acute lymphoblastic leukemia, *MDS* myelodysplastic syndrome, *HL* Hodgkin’s lymphoma, *NHL* non-Hodgkin’s lymphoma, *MM* multiple myeloma, *CML* chronic myeloid leukemia, *CLL* chronic lymphoblastic leukemia. Statistical methods included medians, minimums, and maximums for continuous data and counts and percentages for categorical data. The Mann-Whitney test was used to compare the median values between the groups and the Fisher’s exact test was used to compare categorical data with de-escalation group; a *p* value < 0.05 was considered statistically significant

**Fig. 2:**
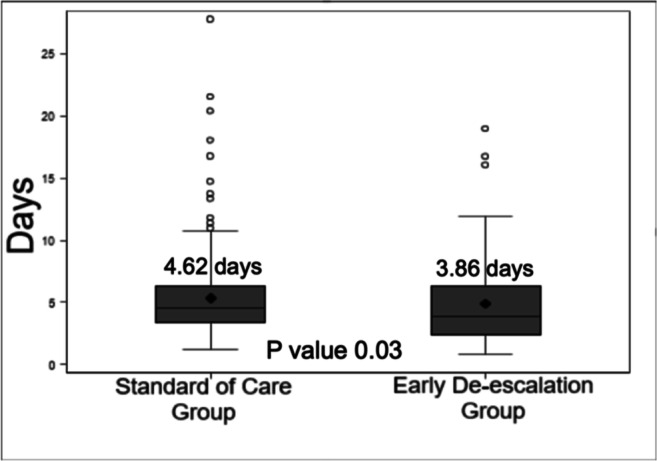
Median duration of BSA use. Primary endpoint demonstrating median duration of BSA utilization was significantly less in the EDG compared to the SCG

The primary endpoint of total duration of BSA within 30 days was significantly less in EDG compared to SCG (median 3.86 vs 4.62 days, *p* = 0.03) (Fig. 2). The median duration of neutropenia after BSA de-escalation was 1.7 days in EDG. Secondary clinical outcomes within 30 days of FN in EDG vs. SCG were not statistically different (Table 2): re-hospitalization rates (EDG 7.2% vs. SCG 10.7% *p* = 0.51), mortality (EDG 0% vs. SCG 0.4% *p* = 1.00), clinical decompensation requiring ICU transfer (EDG 0% vs. SCG 1.4% *p* = 0.56), and pressor use (EDG 0% vs. SCG 0.9% *p* = 1.00).

**Table 2:** Primary and secondary outcomes at 30 days

	Early de-escalation group	Standard of care group	*P* value
Total duration of BSA	3.86	4.62	0.03
LOS from initial FN episode	6.96	6.4	0.048
New infection identified	11 (13.2%)	18 (8.4%)	0.27
Fever recurrences	15 (18%)	18 (8%)	0.02
Clinical de-compensation			
ICU transfer	0	3 (1.4%)	0.56
Pressor use	0	2 (0.9%)	1.00
Re-hospitalization	6 (7.2%)	23 (10.7%)	0.51
Mortality	0	1 (0.4%)	1.00

**Fig. 3:**
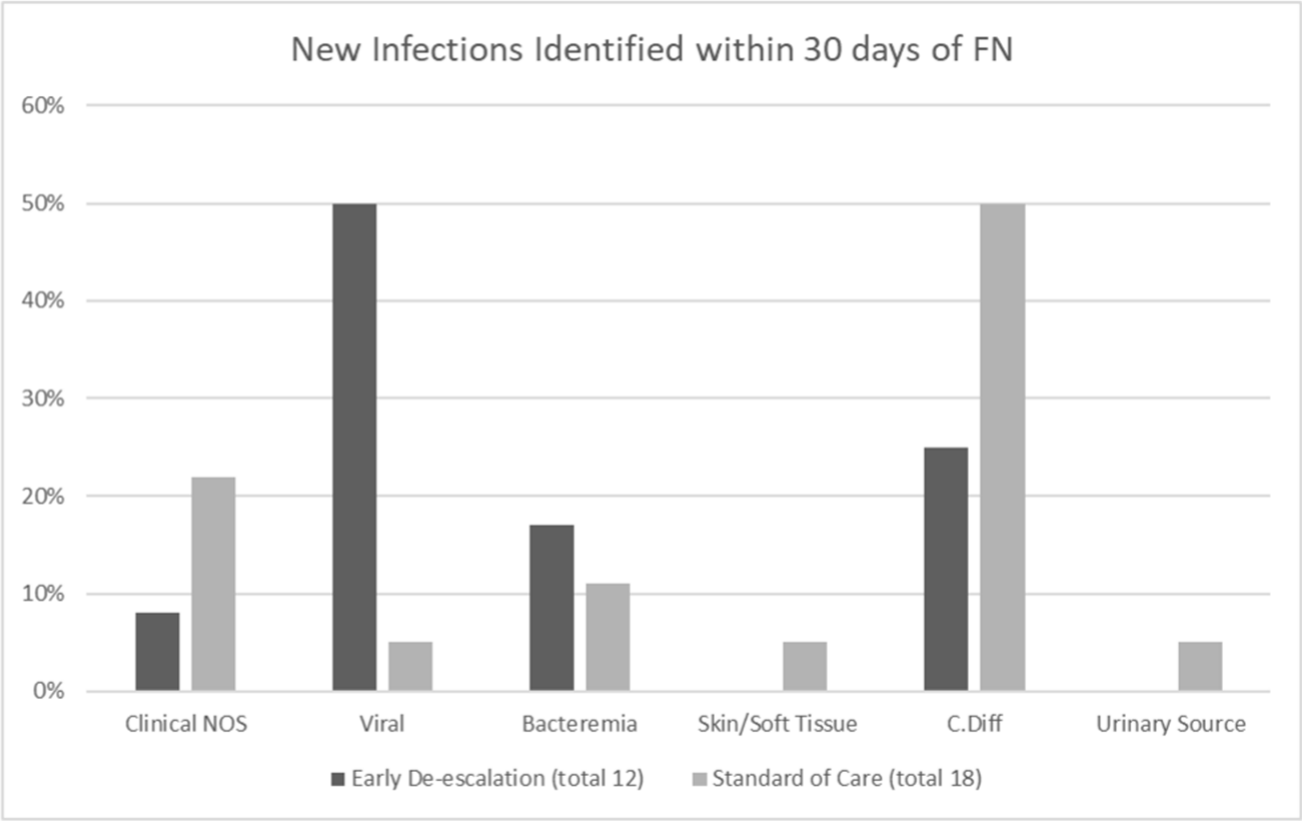
New infections identified within 30 days of FN. New infections identified within 30 days of FN were split into either clinical NOS or microbiologically documented. Clinical NOS included infections identified by clinical symptoms only with no microbiologic data. Two patients in the SCG experienced bacteremia (*Enterococcus faecium* and coagulase-negative *Staphylococcus*. Two patients in EDG experienced bacteremia, one patient had both *Stomatococcus* spp. and *Staphylococcus epidermidis* and one had multi-drug-resistant *Pseudomonas aeruginosa*. One urinary tract infection was identified as *Enterococcus faecalis*. One skin/soft tissue infection was identified as methicillin-sensitive *Staphylococcus aureus*

New infections within 30 days of initial FN were identified in 13.2% of EDG vs. 8.4% SCG (*p* = 0.27). New infections were divided into clinically diagnosed not otherwise specified and microbiologically diagnosed categories (Fig. 3). Of the new microbiologically identified infections, there was a higher incidence of viral infections found in EDG than SCG (7% vs. 0.5% *p* = 0.002) and similar rates of new bacterial infections found in EDG vs. SCG (5% vs. 6% *p* = 0.79). Four patients experienced bacteremia, 2 in SCG and 2 in EDG. Clostridioides difficile (c.diff) was documented in 12 patients, 9 in SCG and 3 in EDG. There was one urinary tract infection and one skin/soft tissue infection in SCG and none of either in EDG.

There was a higher number of fever recurrences (15% vs. 8.4% *p* = 0.023) and subsequently higher rates of BSA re-escalation in EDG (19.2% vs. 8.4% *p* = 0.01). LOS from initial FN was slightly longer in EDG 6.96 days vs. 6.4 days (*p* = 0.048).

## Discussion

This is the largest study of exclusively HSCT patients who had BSA de-escalated while remaining neutropenic. Overall, our study demonstrated fewer days of BSA in EDG without increase in death, re-hospitalization, and clinical de-compensation within 30 days of the initial FN episode. These results are consistent with similar studies (Table 3); however, our findings were uniquely driven by a clearly stated and institutionally applied clinical definition of eligibility for early de-escalation of BSA in the neutropenic HSCT population. We limited the EDG group only to patients who were de-escalated to prophylaxis for at least one hospital day or more prior to neutrophil recovery, so as not to artificially “improve” the efficacy and safety assessments by including those soon-to-recover patients from analysis. Thus, the population evaluated remained neutropenic for more than a day after de-escalation and the results demonstrated that BSA de-escalation in HSCT recipients was safe during this very vulnerable period, with no worse outcomes compared to patients who were continued on BSA until ANC recovery.

**Table 3:** Comparison of outcomes among early de-escalation literature

	UNMC	How Long	Moffitt	ANTIBIOSTOP phase 1	ANTIBIOSTOP phase 2
Study population	HSCT recipients	Hematologic malignancy receiving either therapy or HSCT	Allogeneic HSCT recipients	Chemotherapy-induced neutropenia in hematologic malignancy	Chemotherapy-induced neutropenia in hematologic malignancy
# of patients in ED group	83	78	46	32	30
Most common antibacterial prophylaxis	FQ	Not routinely used	FQ	Amoxicillin	Amoxicillin
Duration of neutropenia	9 days	14 days	18 days	20 days	12 days
Duration of neutropenia from BSA ED	1.7 days	N/A	N/A	3 days	3 days
Fever recurrence	18%	14%	15%	42%	40.5%
New infection post-ED	13.2%	36%	4%^a^	22%	27%
ICU admission	0%	N/A	0%	2.2%	13.5%
Death	0%	1%	0%^b^	2.2%^b^	5.4%
Duration of BSA	3.86 days	11.9 days	8.3 days	7 days	5 days

*FQ* fluoroquinolone, *ED* early de-escalation, *ppx* prophylaxis, *N/A* this value was not reported

^a^Only reported *Clostridium difficile* infections

^b^Reported only in hospital mortality

Several prospective studies have demonstrated safe de-escalation of BSA while high-risk patients are still neutropenic including the HOW LONG study [16] and the ANTIBIOSTOP study [17] as well as a smaller retrospective study from Moffitt and colleagues [18]; however, overall data to support this practice is still sparse. Table 3 compares common outcomes between these data and the current UNMC study, but it is important to note that varying definitions and criteria for BSA de-escalation as well as differing primary endpoints between studies make it difficult to compare them. The HOW LONG study discontinued BSA with resolution of fever for 72 h or more regardless of neutrophil count, but only 53% of patients remained neutropenic at BSA withdrawal in the early de-escalation group [16]. The Moffitt study de-escalated BSA to original prophylactic antimicrobial agents while still neutropenic after at least 5 days of BSA therapy and afebrile for 48 h [18]. However, they did not describe a minimum amount of time the patient was required to be neutropenic to be included in the de-escalation group nor did they report a median duration of neutropenia from de-escalation. The ANTIBIOSTOP study consisted of two phases for early de-escalation: the first phase stopped antibiotics within 48 h of being afebrile while the second phase stopped antibiotics after 5 days of BSA regardless of fever curve. Both groups had a majority of patients who were neutropenic at the cessation of BSA but no standard of care group, thus resulting in similar outcomes between the two groups [17].

We were able to demonstrate in the UNMC study that de-escalation was safe once neutropenic patients with fever of unknown origin were afebrile for at least 48 h on empirical antibiotics and remained hemodynamically stable, even if they had not received a pre-determined course of antibiotics. Both the Moffitt and ANTIBIOSTOP trials required patients to have been on at least 5 days of BSA therapy in their early de-escalation groups, which translated to longer BSA durations overall [17, 18]. Adverse outcomes including ICU admissions and mortality were similar if not lower in our study without a pre-specified BSA course (Fig. 3), though these events were too infrequent to draw definitive conclusions. Kroll et al. also retrospectively looked at BSA de-escalation in patients who remained neutropenic after HSCT, but patients could not be de-escalated until after they had already received 14 days of BSA, bringing into question whether this was truly a de-escalation strategy [19].

Rates of recurrent fevers were higher in EDG, but this was expected and seen commonly across similar studies (Fig. 3). While recrudescence of fever called for re-escalation of BSA in some cases in our study, this additional BSA exposure was accounted for in the primary outcome by calculating the total duration of BSA within 30 days. The EDG also included a higher-risk population overall with significantly more patients undergoing allogeneic HSCTs with acute leukemia as their underlying disease. Despite this higher-risk population having increased incidence of recurrent FN, patients in EDG received significantly lower durations of BSA, indicating that antibiotic de-escalation was not associated with high rates of subsequent bacterial infections, which would require longer durations of antibiotic therapy. In fact, the frequency of new bacterial infections was not statistically significant between the groups in our study. Furthermore, high rates of recurrent FN did not reflect any increase in subsequent clinical decompensation, re-hospitalization, or death within 30 days. This indicated that although patients were likely to develop fevers off antibiotics, recurrent FN alone is not a clinically significant outcome and therefore its risk should not drive duration of BSA use during neutropenia.

Limitations to this study include its single center nature, and as we are a moderate sized HSCT program, its applicability to large programs across the country is not yet known. Notably, while there was not a difference in the rates of multi-drug -resistant bacteria in the new infections identified, there too few infections, and follow-up time was not long enough to adequately assess the impact of de-escalation on the population’s risk for antibiotic resistant. Other limitations include selection bias due to this study’s retrospective design and inability to control for variables such as when to de-escalate antibiotics.

In this high-risk population, antibiotic de-escalation and antimicrobial stewardship strategies are difficult, but a growing body of scientific evidence supports cessation of BSA prior to ANC recovery. Use of empirical antibiotics for FN should be balanced with concerns for growing bacterial resistance [20, 21], disruptions in normal gut flora [21–24], and increasing rates of antibiotic adverse drug events, including *C. difficile* infections [25]. Further prospective, multi-center-randomized trials in both the autologous and allogeneic HSCT population are essential to provide more solid answers about the safety and efficacy of de-escalation in an era when antibiotic overuse is a major problem.

In summary, early de-escalation of empiric antibiotics for FN to levofloxacin prophylaxis is a safe management option for allogeneic or autologous HSCT recipients during neutropenia who are clinically stable, remain afebrile for at least 48 h, and with no documented infection. Implications from this study demonstrating safe early de-escalation of BSA will help guide antimicrobial stewardship programs in this clinical scenario and likely be able to decrease antibiotic days more than has been demonstrated in this and other studies. These findings may provide a model for similar institutions that seek to decrease BSA usage in the HSCT population during FN episodes.
